# Total vascular resistance, augmentation index, and augmentation pressure increase in patients with peripheral artery disease

**DOI:** 10.1097/MD.0000000000026931

**Published:** 2021-08-13

**Authors:** Rika Takemoto, Haruhito A. Uchida, Hironobu Toda, Ken Okada, Fumio Otsuka, Hiroshi Ito, Jun Wada

**Affiliations:** a Department of Nephrology, Rheumatology, Endocrinology and Metabolism, Okayama University Graduate School of Medicine, Dentistry and Pharmaceutical Sciences, Okayama, Japan; b Ultrasound Diagnostics Center, Okayama University Hospital, Okayama, Japan; c Department of Chronic Kidney Disease and Cardiovascular Disease, Okayama University Graduate School of Medicine, Dentistry and Pharmaceutical Sciences, Okayama, Japan; d Department of Cardiovascular Medicine, Okayama University Graduate School of Medicine, Dentistry and Pharmaceutical Sciences, Okayama, Japan; e Department of Medical Support, Okayama University Hospital, Okayama, Japan; f Department of General Medicine, Okayama University Graduate School of Medicine, Dentistry and Pharmaceutical Sciences, Okayama, Japan.

**Keywords:** augmentation index, augmentation pressure, peripheral arterial disease, total vascular resistance

## Abstract

Peripheral arterial disease (PAD) is one of major vascular diseases which frequently coexists with coronary arterial disease and cerebrovascular disease. The patients with PAD have a poor prognosis when it progresses. A new blood pressure testing device enables to simultaneously measure brachial blood pressure (BP), central BP, and several vascular parameters, with easy and non-invasive, in a short time. Here, we aimed to evaluate these arterial stiffness parameters in patients with PAD.

In this study, 243 consecutive patients who were suspected of having PAD and referred to our hospital from September 2016 to June 2019, were registered. Several parameters, such as brachial BP, central BP, aortic pulse wave velocity (aPWV), total vascular resistance (TVR), augmentation index (AI) and augmentation pressure (AP), were determined by Mobil-O-Graph. Ankle-brachial pressure index (ABI) was used to define PAD (ABI ≤ 0.9 as PAD). The relationship between PAD and central BP, aPWV, TVR, AI, or AP were investigated.

One hundred sixty-two patients (67%) were categorized as the PAD group and 81 patients (33%) as the non-PAD group. In the PAD group, the systolic brachial BP and central systolic BP were significantly higher than those in the non-PAD group (138 ± 24 mmHg vs 131 ± 19 mmHg, *P* < .05, 125 ± 22 mmHg vs 119 ± 18 mmHg, *P* < .05, respectively). TVR, AI, and AP were significantly higher in the PAD group (1785 ± 379 dyn s/cm^5^ vs 1661 ± 317 dyn s/cm^5^, *P* < .05, 26.2 ± 13.0% vs 22.2 ± 13.3%, *P* < .05, 13.5 ± 9.4 mmHg vs 10.7 ± 7.2 mmHg, *P* < .05, respectively). No significant differences in diastolic BP, central diastolic BP, and aPWV were found between the groups. Multivariate logistic regression analysis revealed that PAD was significantly associated with TVR, AI, and AP (*P* < .05, respectively).

TVR/AP/AI were significantly higher in the PAD group than in the non-PAD group.

## 1 Introduction

In recent years, the increase of lifestyle-related diseases largely associated with metabolic syndrome has been remarkable.^[1–3]^ Along with this increment, arteriosclerotic diseases are also increasing.^[1,2]^ Atherosclerosis can occur in blood vessels throughout the body. Peripheral vascular disease (PAD) is a condition in which arteriosclerosis mainly occurs in blood vessels of the limbs and is usually determined by ankle-brachial pressure index (ABI).^[1,2,4,5]^ The main causes of PAD are lifestyle-related diseases, such as hypertension, diabetes, dyslipidemia, chronic kidney disease, smoking, and obesity. It is well known that the cause of increased mortality when PAD progresses to foot amputation.^[1,4]^

Arterial stiffness can be assessed by a variety of methods. Increase of arterial stiffness is associated with higher risk for the development of arteriosclerosis and recognized as a prognostic marker beyond standard risk factors. Currently, arterial stiffness is able to be measured non-invasively and reproducibly.^[6–9]^

To date, to evaluate vascular dysfunction, an early stage of arteriosclerosis, several devises have been developed. Pulse wave velocity (PWV) is widely used in clinical practice as a parameter for arterial stiffness.^[10,11]^ Flow mediated dilatation represents vascular endothelial function.^[12,13]^ Cardio-ankle vascular index is used for estimating the extent of arterial stiffness and atherosclerosis.^[14–16]^ Augmentation index is used as a surrogate measure of arterial stiffness.^[17–19]^ Multiple measurements for these parameters for evaluation of vascular function provide a clue of arteriosclerosis. These parameters are widely used in clinical practice worldwide, however, the accuracy of these methods is unsatisfactory especially in the patients with PAD. Vascular catheterization can evaluate hemodynamics in patients with PAD, such as vascular resistance and arterial central blood pressure, but invasive. Recently, Mobil-O-graph has been developed and it enables non-invasive and simultaneous measurement of several vascular parameters with shorter procedure duration.^[20,21]^ So far, the evaluation of PAD using Mobil-O-graph has not been reported.

Therefore, in this study, we aimed to examine the association of several parameters determined by Mobil-O-graph, such as central blood pressure, brachial blood pressure, aortic PWV (aPWV), total vascular resistance (TVR), augmentation pressure (AP), and augmentation index (AI), with PAD. Among them, TVR, AP, and AI were found to have a significant association with PAD.

## 2 Materials and methods

### 2.1 Study design

This was a single-center, and cross-sectional observational study.

### 2.2 Participants

The participants were recruited from outpatients who were suspected of having PAD and referred to our hospital from September 2016 to June 2019, and who were 20 years old and over, and obtained informed consent of the present study. Patients who disagreed to participate in this study were excluded.

Based on physician's charts, characteristics of each participant was evaluated, including age, sex, body mass index, smoking, diabetes mellitus, hypertension, dyslipidemia, chronic kidney disease, ischemic heart disease, cerebral infarction, and medications. Lab data were collected as following: serum creatinine, calcium, phosphorus, hemoglobin, fasting plasma glucose, total cholesterol, triglycerides, high-density lipoprotein cholesterol, and low-density lipoprotein cholesterol.

Participants received a test under fasting, alcohol-free, and non-smoking, or refraining smoking at least 12 hours condition.

The definition of disease is as follows; diabetes mellitus was defined by receiving medication for diabetes mellitus or fulfilling the diagnostic criteria: postprandial plasma glucose levels ≥200 mg/dL and/or HbA1c ≥6.5%. Dyslipidemia was defined by receiving medication for dyslipidemia or fulfilling the diagnostic criteria: LDL-cholesterol ≥140 mg/dL, HDL-cholesterol <40 mg/dL, triglycerides ≥150 mg/dL. Chronic kidney disease was defined as eGFR <60 mL/min/1.73 m^2^ or urinary protein≥±. In the dipstick test, ± is considered equal to 0.15 g/gCr.

Hypertension was defined by receiving anti-hypertensive agents or ≥140/90 mmHg. Ischemic heart disease was defined as symptomatic and having history of percutaneous coronary intervention treatment and/or surgical operation of coronary artery bypass grafting. Cerebral infarction was defined as symptomatic and requiring hospital treatment. Smoking included both current and past habitat of smoking.

### 2.3 Evaluation of ankle-brachial pressure index

ABI was determined with BP-203RPE II form (FUKUDA COLIN, Japan) or VaSera VS-1500A (FUKUDA DENSHI, Japan), followed manufactural instruction. In brief, ABI was measured after resting in a supine position for 5 minutes in a temperature-controlled (22–25 °C) room. Blood pressure was measured using oscillometric method. Oscillometry was based on the assumption that maximum oscillations occurring in the vessel in slow cuff deflation correspond to mean arterial pressure from which mathematical formulas can deduct the systolic and diastolic blood pressure.^[22,23]^ Cuffs were applied to both upper arms and ankles. ABI was calculated dividing the ankle SBP in each leg by the higher SBP of upper arm. The lower ABI value was used as representative ABI of each participant.

### 2.4 Definition of the peripheral artery disease

PAD was defined as ABI ≤0.9.

### 2.5 Evaluation measurements of vascular parameter

Several vascular parameters were measured using Mobil-O-Graph (IEM, Stolberg, Germany); brachial systolic blood pressure (bSBP), brachial diastolic blood pressure, brachial pulse pressure (bPP), central systolic blood pressure (cSBP), central diastolic blood pressure, central pulse pressure (cPP), aPWV, TVR, AP, AI, stroke volume, cardiac output, and cardiac index. Each participant laid on the bed in a spine position for >5 minutes in a quiet and temperature-controlled room, then, the measurement was performed in accordance with the manufacturer instruction.

The Mobil-O-Graph had an inbuilt ARCSolver (Austrian Institute of Technology, Vienna, Austria). After brachial BP was measured, the cuff was instantly inflated, and brachial artery pressure waves were recorded, holding the diastolic BP level for 10 seconds. Brachial artery pressure wave was digitized with a high-fidelity pressure sensor connected to a 12-bit A/D converter. Central AI and AP were calculated using a generalized transfer function. The ARCSolver transfer function includes an algorithm for checking the signal quality. We analyzed only excellent or good-quality results, that is, >80% or >50% of signals used for the transfer function, respectively.^[24]^

This running 1st pulse wave gets reflected from distal branching point of aortic wall and generates 2nd reflected wave. Arterial stiffness mainly determines the morphology of reflected 2nd wave. AIX and PWV were obtained with the help of algorithm and mathematical model in the inbuilt software by using amplitude and time difference of 1st and 2nd wave.^[25]^

### 2.6 Ethics

This study followed the Declaration of Helsinki (seventh revision, 2013) on medical protocol and ethics. Informed consent was obtained from all participants. The ethics committee of the Institutional Review Board in Okayama University Hospital approved the protocol (Ken1609-029).

### 2.7 Statistics

All data are presented as the mean ± standard deviation or number (%). Differences between the 2 groups were examined by a student *t* test or chi-squared test where appropriate. Multivariate logistic regression analyses were performed to investigate the association of TVR, AP, and AI with PAD. Statistical significance was defined as *P* < .05. All data were analyzed using JMP (version 13.0, SAS Institute Inc., Cary, NC).

## 3 Results

### 3.1 Characteristics of the participants

Characteristics of the participants are shown in Table 1. A total of 243 participants were enrolled. One hundred sixty-two patients (67%) were categorized as the PAD group and 81 patients (33%) as the non-PAD group. No significant differences were found in baseline characteristics between the groups, except for smoking habitat (*P* = .0272) and usage of antiplatelet drugs (*P* = .0007).

**Table 1:** Characteristic of the study participants.

Characteristic	n = 243	PAD (n = 162)	Non-PAD (n = 81)	*P* value
Age, yr	71 ± 10	72 ± 11	70 ± 10	.5585
Sex (male, %)	165 (68)	108 (65)	54 (69)	.2452
Body mass index, kg/m^2^	22.5 ± 4.0	22.2 ± 3.8	23.0 ± 4.3	.1794
Smoking (%)	183 (75)	129 (80)	54 (67)	.0272
Diabetes mellitus (%)	141 (58)	97 (60)	44 (54)	.4081
Hypertension (%)	209 (86)	141 (87)	68 (84)	.5132
Dyslipidemia (%)	176 (72)	121 (75)	55 (68)	.2642
Chronic kidney disease (%)	155 (64)	108 (67)	47 (58)	.1864
Ischemic heart disease (%)	75 (31)	51 (31)	24 (30)	.7683
Cerebral infarction (%)	36 (15)	26 (16)	10 (12)	.4436
Medications				
Antiplatelet agent (%)	172 (71)	126 (78)	46 (57)	.0007
Statin (%)	123 (51)	81 (50)	42 (52)	.7855
ACEI (%)	33 (14)	22 (14)	11 (14)	1.0000
ARB (%)	109 (45)	77 (48)	32 (40)	.2358
β-blocker (%)	94 (39)	66 (41)	28 (35)	.3517
CCB (%)	134 (55)	88 (54)	46 (57)	.7152
Diuretics (%)	65 (27)	45 (28)	20 (25)	.6084
Anticoagulant (%)	49 (20)	28 (17)	21 (26)	.1135
Lab data				
Creatinine, mg/dL	2.16 ± 3.02	2.27 ± 3.10	1.93 ± 2.87	.3987
Calcium, mg/dL	9.1 ± 0.6	9.1 ± 0.6	9.0 ± 0.48	.3134
Phosphorus, mg/dL	3.8 ± 1.0	3.8 ± 1.0	3.9 ± 1.2	.6177
Hemoglobin, g/dL	12.5 ± 1.9	12.6 ± 1.9	12.3 ± 1.9	.3144
Fasting plasma glucose, mg/dL	115 ± 35	114 ± 38	117 ± 27	.5946
Total cholesterol, mg/dL	117 ± 36	178 ± 37	176 ± 36	.7477
Triglycerides, mg/dL	155 ± 121	150 ± 102	167 ± 155	.4253
High-density lipoprotein cholesterol, mg/dL	53 ± 16	53 ± 17	53 ± 15	.8750
Low-density lipoprotein cholesterol, mg/dL	101 ± 31	100 ± 31	102 ± 32	.7022

### 3.2 Vascular parameters of participants

Vascular parameters are shown in Table 2. In the PAD group, the bSBP, cSBP, bPP, and cPP were significantly higher than those in the non-PAD group (138 ± 24 mmHg vs 131 ± 19 mmHg, *P* = .0129, 125 ± 22 mmHg vs 119 ± 18 mmHg, *P* = .0295, 57 ± 17 mmHg vs 51 ± 12 mmHg, *P* = .0047, 42 ± 15 mmHg vs 38 ± 11 mmHg, *P* = .0260, respectively). TVR, AI, and AP were significantly higher in the PAD group (1785 ± 379 dyn s/cm^5^ vs 1661 ± 317 dyn s/cm^5^, *P* = .0079, 26.2 ± 13.0% vs 22.2 ± 13.3%, *P* = .0284, 13.5 ± 9.4 mmHg vs 10.7 ± 7.2 mmHg, *P* = .0103, respectively). No significant differences in brachial diastolic blood pressure, central diastolic blood pressure, aPWV, stroke volume, cardiac output, nor cardiac index, between the groups.

**Table 2:** Measurements by Mobil-O-Graph.

	n = 243	PAD (n = 162)	Non-PAD (n = 81)	*P* value
bSBP, mmHg	136 ± 23	138 ± 24	131 ± 19	.0129
bDBP, mmHg	81 ± 12	81 ± 13	80 ± 12	.3602
bPP, mmHg	55 ± 16	57 ± 17	51 ± 12	.0047
cSBP, mmHg	123 ± 21	125 ± 22	119 ± 18	.0295
cDBP, mmHg	83 ± 13	83 ± 13	81 ± 12	.2937
cPP, mmHg	41 ± 14	42 ± 15	38 ± 11	.0260
aPWV, m/s	10.8 ± 2.0	10.9 ± 2	10.5 ± 2	.0694
Total vascular resistance, dyn s/cm^5^	1744 ± 364	1785 ± 379	1661 ± 317	.0079
Augmentation index (%)	24.9 ± 13.2	26.2 ± 13	22.2 ± 13.3	.0284
Augmentation pressure, mmHg	12.6 ± 8.8	13.5 ± 9.4	10.7 ± 7.2	.0103
Stroke volume, mL	73 ± 14	72 ± 13	75 ± 15	.0826
Cardiac output, L/min	5.0 ± 0.9	4.9 ± 0.9	5.1 ± 0.9	.2093
Cardiac index, L/min/m^2^	3.1 ± 0.7	3.1 ± 0.7	3.1 ± 0.7	.9649

### 3.3 Multivariate logistic regression analyses for TVR, AP, and AI

Multivariate logistic regression analyses were performed to investigate the association of PAD with TVR, AP, and AI. As shown in Table 3 model 1, the results demonstrated that the PAD was significantly associated with TVR (OR: 8.2003, 95% CI: 1.5084–49.5750, *P* = .0178), as well as AP and AI (*P* = .0316, *P* = .0327, respectively) adjusted with age, sex, and body mass index. These significant association remained adjusted with model 1 covariates plus smoking (Table 3, model 2). In model 3, further adjusted by classical risk factors for cardiovascular diseases, the association between PAD and TVR still remained significant (OR: 5.7677, 95% CI: 1.0028–36.8522, *P* = .0496).

**Table 3:** Multivariate analysis for TVR, AP, and AI.

	Odds ratio	95% CI	*P*
TVR			
Model 1	8.2003	1.5084–49.5750	.0178
Model 2	7.4557	1.3218–46.7984	.0267
Model 3	5.7677	1.0028–36.8522	.0496
AP			
Model 1	5.5435	1.1632–26.4088	.0316
Model 2	5.1042	1.1002–27.0629	.0451
Model 3	4.0939	0.8490–22.4330	.0901
AI			
Model 1	4.8282	1.1504–20.8968	.0327
Model 2	4.8889	1.1218–21.9658	.0359
Model 3	4.3908	0.9861–20.0614	.0534

## 4 Discussion

In this study, we found that bSBP, cSBP, bPP, and cPP in the PAD group were significantly higher than those in the non-PAD group. In addition, TVR, AI, and AP were significantly higher in the PAD group compared with non-PAD group. Moreover, a multivariate logistic regression analyses demonstrated that PAD was significantly associated with TVR, AP, and AI adjusted with established multiple risk factors.

TVR basically presents resistance to blood flow that occurs in blood vessels. The definition of TVR is calculated as follows; TVR is equal to mean arterial BP divided by cardiac output. TVR is significantly higher in women than in men.^[26]^ Especially, elevated TVR was observed in older women.^[26]^ It is also reported that high TVR is associated with mortality, heart failure, and CVD events.^[25]^ Thus, increased TVR may further enhance the accumulated risk for cardiovascular disease. As expected, in the current study, higher TVR is associated with the presence of PAD. This might be simply because the risks for PAD are common compared with other heart and vascular diseases such as heart failure, ischemic heart disease, and stroke. Indeed, the patients with PAD are known to have high CAD risk.^[1,27]^ The other reason is that the mean arterial BP elevated in patients with arterial sclerosis and several patients with PAD have a low cardiac output due to CAD. Consequently, TVR may increase by reflecting peripheral arterial stenosis and low cardiac output. Therefore, TVR well-represents the vascular condition of atherosclerosis throughout the body.

AP represents changes in central hemodynamics and pressure wave characteristics resulting from increased aortic wall stiffening and aortic remodeling.^[11]^ The central AP determined by the Mobil-O-Graph was reported to be associated with age and sex in the European community-based population.^[28]^ Higher central AP is associated with increased risk of development of CAD in younger and middle-aged men.^[28]^ Thus, AP may reflect the accumulation of cardiovascular risks in association with aortic wall stiffening. In the current study, AP in the PAD group is significantly higher than that in the non-PAD group. aPWV is also known as one of the surrogate markers for aortic stiffness.^[25]^ However, our study showed no difference in aPWV between the PAD group and the non-PAD groups. This result suggests that aPWV may not reflect the peripheral vascular condition but represents central vascular condition. AP may be a better way to evaluate arterial stiffness than aPWV.

AI presents an indirect measurement of arterial stiffness.^[29,30]^ Several papers have reported the clinical significance of AI. An increase in central AI is related to a risk of increase of cardiovascular events and all-cause mortality.^[11,31]^ AI can predict clinical cardiovascular events independent of peripheral pressures.^[11]^ AI is recognized as one of useful markers of arterial stiffness in patients with PAD.^[32]^ AI is higher in patients with PAD as compared with control patients.^[33]^ In consistent with these studies, AI in the PAD group is significantly higher than that in non-PAD group in the present study, although all participants were referred to our institute to be suspicious for PAD. This result suggests that higher value of AI may be linked to vascular stenosis or occlusion in the peripheral artery. In this sense, AI can represent from mild to severe vascular condition.

Several limitations in this study are noted. Firstly, this study was conducted in a single-institution study, with a small number of participants. Secondly, since all participants were referred to our hospital to be suspicious for PAD, the background of all participants were bias. That is, all participants in this study had, at least one or more risks for atherosclerosis. No healthy person was examined. Future study will be required to conduct a study in the general population. Third, since the current study was a cross-sectional observation study, the effect of the interventional treatments for PAD on the changes in these parameters were not clarified. Fourth, in this study, PAD was defined only by ABI value, however, ABI alone cannot provide a precise diagnosis of PAD in clinical setting. Fifth, in the present study, ABI value was determined by the oscillometric method, not by the doppler method. Future study will be required to further confirm whether TVR/AP/AI are valuable markers for PAD.

## 5 Conclusions

TVR, AP, and AI were significantly higher in the PAD group than in the non-PAD group. Non-invasive, easy, and reproducible tool is desirable to assess arterial stiffness.

## Acknowledgments

The authors would like to thank Dr. Hiroaki Otsuka and Dr. Kentaro Ejiri from Department of Cardiovascular Medicine, Okayama University Graduate School of Medicine, Dentistry and Pharmaceutical Sciences for patient recruitments, and Dr. Shugo Okamoto, Dr. Yasuhiro Onishi, Dr. Mariko Tsuchida-Nishiwaki, Dr. Natsumi Matsuoka-Uchiyama, Dr. Nozomu Otaka, Dr. Yuki Kakio, Dr. Hidemi Takeuchi, and Dr. Yoshiko Hada from Department of Nephrology, Rheumatology, Endocrinology and Metabolism, Okayama University Graduate School of Medicine, Dentistry and Pharmaceutical Sciences for helpful comment.

## Author contributions

**Conceptualization:** Rika Takemoto, Haruhito A. Uchida.

**Data curation:** Rika Takemoto.

**Formal analysis:** Rika Takemoto, Haruhito A. Uchida.

**Investigation:** Rika Takemoto, Haruhito A. Uchida, Hironobu Toda.

**Writing – original draft:** Rika Takemoto.

**Writing – review & editing:** Haruhito A. Uchida, Hironobu Toda, Ken Okada, Fumio Otsuka, Hiroshi Ito, Jun Wada.
